# Potassium Channels and Human Epileptic Phenotypes: An Updated Overview

**DOI:** 10.3389/fncel.2016.00081

**Published:** 2016-03-30

**Authors:** Chiara Villa, Romina Combi

**Affiliations:** School of Medicine and Surgery, University of Milano-BicoccaMonza, Italy

**Keywords:** K^+^ channels, epilepsy, mutation, KCNT1, Kir channels, Kv channels

## Abstract

Potassium (K^+^) channels are expressed in almost every cells and are ubiquitous in neuronal and glial cell membranes. These channels have been implicated in different disorders, in particular in epilepsy. K^+^ channel diversity depends on the presence in the human genome of a large number of genes either encoding pore-forming or accessory subunits. More than 80 genes encoding the K^+^ channels were cloned and they represent the largest group of ion channels regulating the electrical activity of cells in different tissues, including the brain. It is therefore not surprising that mutations in these genes lead to K^+^ channels dysfunctions linked to inherited epilepsy in humans and non-human model animals. This article reviews genetic and molecular progresses in exploring the pathogenesis of different human epilepsies, with special emphasis on the role of K^+^ channels in monogenic forms.

## Introduction

Epilepsy is one of the most common neurological disorders characterized by abnormal electrical activity in the central nervous system (CNS) and recurrent seizures represent a cardinal clinical manifestation. The phenotypic expression of each seizure is determined by the original point of the hyperexcitability and its degree of spread in the brain (Steinlein, 2004). Several brain defects due to membrane instability could cause epilepsy.

In the last two decades, gene defects underlying different forms of epilepsy have been identified and most of these genes code for ion channels, which thus appear as important players in the etiopathogenesis of idiopathic epilepsy. Indeed, several epileptic phenotypes have been associated to dysfunctions of potassium (K^+^) channels (Brenner and Wilcox, 2012). It has been recently proposed to name such epilepsies as “K^+^ channelepsies” (D’Adamo et al., 2013). These channels play a major role in neuronal excitability and their importance is related to the level of their expression in subcellular domain, individual cell, or circuit (Cooper, 2012). K^+^ channels are also involved in setting the inward-negative resting membrane potential. Based on their structures, biophysical characteristics, pharmacological sensitivities and physiology, these channels are classified as voltage-gated (Kv), inwardly rectifying (Kir), sodium (Na)-activated channels or Ca^2+^-activated channels (**Table 1**; González et al., 2012).

**Table 1:** Summary of human K^+^ channels subfamilies involved in epilepsies.

Subfamily	Main functions	Cloned subunits	Subunits associated with epilepsy
Voltage-gated K^+^ channels (Kv)	Regulation of outward K^+^ currents and action potentials, modulation of neurotransmitter release, control of both excitability and electrical properties of neurons	Kv1-12	Kv1.1; Kv1.2
			Kv4.2; Kv4.3
			Kv7.1; Kv7.2; Kv7.3
			Kv8.2
			Kv11.1

Inwardly rectifying K^+^ channels (Kir)	Maintenance of the resting membrane potentials and regulation of the cell excitability	Kir1-7	Kir2.1
			Kir4.1
			Kir6.2

Sodium-activated K^+^ channels (K_Na_)	Regulation of delayed outward currents *I*_KNa_ and contribution to adaptation of firing rate	K_Ca4.1_ (Slack) K_Ca4.2_ (Slick)	K_Ca4.1_ (Slack)

Calcium-activated K^+^ channels (K_Ca_)	Regulation of neuronal firing properties and circuit excitability	K_Ca1-3_	K_Ca1.1_
		K_Ca5.1_	


Herein we report an updated discussion on the role of mutations in K^+^ channels (**Table 2**) in the pathogenesis of human epilepsy.

**Table 2:** Mutations in K^+^ channels associated with human epileptic phenotypes.

Gene/protein	Epileptic phenotypes	Mutations	Effects on channel functionality	Reference
*KCNA1*/Kv1.1	Generalized or partial seizures associated to EA1	Several heterozygous point mutations	Loss-of-function mutations altering the channel’s properties and frequently associated with reduced currents	Browne et al., 1994; Adelman et al., 1995; D’Adamo et al., 1999; Spauschus et al., 1999; Zuberi et al., 1999; Eunson et al., 2000; Imbrici et al., 2006;

*KCNA2*/Kv1.2	Mild to severe epileptic encephalopathy	p.Ile263Thr	Loss-of-function	Syrbe et al., 2015
		p.Arg297Gln	Gain-of-function		
		p.Leu298Phe	Gain-of-function		
		p.Pro405Leu	Loss-of-function		
	
	Ataxia and myoclonic epilepsy	p.Arg297Gln	Functional analysis are under way	Pena and Coimbra, 2015

*KCND2*/Kv4.2	TLE	p.Asn587fsX1	Channel haploinsufficiency due to truncated Kv4.2 subunit	Singh et al., 2006
	
	Autism and severe intractable seizures	p.Val404Met	Gain-of-function mutation showing	Lee et al., 2014
			slowed channel inactivation	

*KCND3*/Kv4.3	Early onset cerebellar ataxia, intellectual disability, oral apraxia and epilepsy	p.Arg293_Phe295dup	Strong shift of the voltage-dependence of activation and inactivation of the channel subunit	Smets et al., 2015

*KCNQ1*/Kv7.1	LQTS and epilepsy	p.Leu273Phe	No available data on channel functionality	Tiron et al., 2015
	
	SUDEP	p.Ala46Thr	p.Ala46Thr: activation of more rapid current without initial delay	Yang et al., 2009; Partemi et al., 2015
		p.Val287Met		
		p.Val648Ile		

*KCNQ2*/Kv7.2	BFNS with normal cognition or EOEEs with mental retardation	> 80 mutations (missense, non-sense, truncations, splice-site defects, frame-shift mutations, sub-microscopic deletions or duplications)	Impairment of channel function, leading to reduced current densities	Singh et al., 1998; Weckhuysen et al., 2012; Soldovieri et al., 2014

*KCNQ3*/Kv7.3	BFNS with variable age of onset and good outcome	p.Gly340Val	No available data on channel functionality	Zara et al., 2013; Grinton et al., 2015
		p.Arg780Cys		
	
	Early-onset epilepsy and neurocognitive deficits	p.Ile317Thr	Impairment of channel function, leading to reduced current densities	Soldovieri et al., 2014; Miceli et al., 2015
		p.Arg330Leu		
	
	BECTS	p.Arg364His	No available data on channel functionality	Fusco et al., 2015

*KCNV2*/Kv8.2	Febrile and afebrile partial seizures	p.Arg7Lys	Decrease in delayed rectifier K^+^ current in neurons	Jorge et al., 2011
	
	Epileptic encephalopathy and severe refractory epilepsy	p.Met285Arg	Decrease in delayed rectifier K^+^ current in neurons and impairment of the voltage-dependence of the channel	Jorge et al., 2011

*KCNH2*/Kv11.1	Epilepsy associated with LQT2	p.Ile82Thr	Loss-of-function mutations, leading to reduced currents	Keller et al., 2009; Omichi et al., 2010; Tu et al., 2011; Zamorano-León et al., 2012; Partemi et al., 2013
		p.Arg176Trp		
		p.Thr74ArgfsTer32		
		p.Ala429Pro		
		p.Tyr493Phe		
		p.Arg534Cys		
		p.Arg863X		
		p.Arg1047Leu		

*KCNAB2*/Kvβ2	Severe epilepsy	hemizygous deletion of *KCNAB2*	Loss-of-function mutations/ haploinsufficiency	Heilstedt et al., 2001

*LGI1*	ADLTE	> 30 mutations (missense, splice-site mutations, short indels, single microdeletion)	Failure in preventing channel inactivation resulting in more rapidly closing channels	Kalachikov et al., 2002; Morante-Redolat et al., 2002; Nobile et al., 2009; Fanciulli et al., 2012; Dazzo et al., 2015

*KCNJ2*/Kir2.1	Seizures associated to ATS	p.Arg67Gln	Loss-of-function mutations with dominant-negative effects	Haruna et al., 2007; Chan et al., 2010
		p.Gly146Ser		
		p.Thr192Ile		
	
	SQT3s and autism-epilepsy phenotype	p.Lys346Thr	Gain-of-function mutation leading to enhance the channel’s surface expression and stability at the plasma membrane, reduce protein degradation and alter protein compartmentalization	Ambrosini et al., 2014

*KCNJ10*/Kir4.1	Seizure susceptibility	p.Arg271Cys	No observable changes in channel function or in predicted channel structure	Buono et al., 2004; Shang et al., 2005
	
	Epilepsy associated to EAST or SeSAME syndrome	p.Arg65Cys	Loss-of-function recessive mutations	Bockenhauer et al., 2009; Scholl et al., 2009; Freudenthal et al., 2011
		p.Arg65Pro		
		p.Phe75Lys		
		p.Gly77Arg		
		p.Val259fs259X		
	
	Epilepsy associated to ASDs and intellectual disability	p.Arg18Gln p.Val84Met	Gain-of-function mutations leading to increased channel surface expression	Sicca et al., 2011

*KCNJ11*/Kir6.2 *ABCC8*/SUR1	DEND syndrome	Several point mutations	Gain-of-function mutations causing reduction in ATP sensitivity, leading to an increase in the K_ATP_ current	Hattersley and Ashcroft, 2005

*KCNT1*/Slack	ADNFLE associated to intellectual disability and psychiatric features	p.Gly288Ser	Gain-of-function mutations, increasing the cooperativity in channel gating	Heron et al., 2012; Kim et al., 2014; Møller et al., 2015
		p.Arg398Gln		
		p.Tyr796His		
		p.Met896Ile p.Arg928Cys		
	
	EIMFS	p.Val271Phe		Barcia et al., 2012; Ishii et al., 2013; Kim et al., 2014; Mikati et al., 2015; Møller et al., 2015; Ohba et al., 2015; Rizzo et al., 2016
		p.Gly288Ser	Gain-of-function mutations, increasing the cooperativity in channel gating	
		p.Arg398Gln		
		p.Arg428Gln		
		p.Arg474His		
		p.Met516Val		
		p.Lys629Asn		
		p.Ile760Met		
		p.Pro924Leu		
		p.Ala934Thr		
	
	EOEEs	p.Phe932Ile	No available data on channel functionality	Vanderver et al., 2014
	
	OS	p.Ala966Thr	Gain-of-function mutation	Martin et al., 2014; Kim et al., 2014

*KCNMA1*/K_Ca1.1_	Generalized epilepsy and paroxysmal dyskinesia	p.Asp434Gly	Gain-of-function mutation leading to an increase of channel opening probability and Ca^2+^ dependence	Du et al., 2005; Lee and Cui, 2009

*KCNMB3*/ K_ Caβ3_	Idiopathic generalized epilepsy	p.Val256TyrfsTer4	Loss-of-function truncation mutation affecting synaptic inhibition and increasing neuronal excitability	Hu et al., 2003; Lorenz et al., 2007


## Voltage-Gated K^+^ Channels (Kv)

The Kv channels are widely expressed both in the central and in peripheral nervous system where they are involved in several processes (e.g., the regulation of the duration of action potentials, the modulation of the neurotransmitter release, the control of the electrical properties and the firing of neurons). Kv channels generally regulate outward K^+^ currents that contribute to membrane repolarization and hyperpolarization, thus limiting the neuronal excitability. Moreover, they actively participate in cellular and molecular signaling pathways that regulate the life and death of neurons, such as apoptosis, channel phosphorylation, or cell proliferation (Shah and Aizenman, 2014). In particular, neuronal cell apoptosis is correlated to an increased expression of Kv channels at the plasma membrane, thus facilitating more K^+^ efflux and a loss of cytosolic K^+^. This drop in the intracellular K^+^ concentration actives pro-apoptotic enzymes, such as nuclease or caspase that can trigger downstream apoptotic signals culminating in DNA fragmentation or degradation (Leung, 2010).

In human genome, forty different genes encoding for Kv channels were reported and subdivided into twelve sub-families (Kv1 through Kv12) (Gutman et al., 2005). Mammalian Kv channels are tetramers, composed of α-subunits that line an ion pore. Each α-subunit shows six α-helical transmembrane domains (S1–S6), a membrane-reentering P loop between S5 and S6, and cytosolic N-, and C-termini. The S5-P-S6 segments constitute the ion conduction pore, while the S1–S4 sequences are critical for the voltage-sensing and gating of the channel (Brenner and Wilcox, 2012).

Furthermore, α-subunits can bind to regulatory β subunits (Kvβ1, Kvβ2, and Kvβ3) as well as to other Kv channel-interacting proteins. This variability in the channel interactions results in strong modifications of the channel properties (McKeown et al., 2008).

The following Kv subfamilies have been associated with either epilepsy or other disorders showing seizures.

### Kv1

The Kv1 subfamily plays an essential role in the initiation and shaping of action potentials. These channels are expressed at the soma, axons, synaptic terminals, and proximal dendrites. The most abundant Kv1 α-subunits are Kv1.1, Kv1.2, and Kv1.4. These subunits are differentially expressed and their composition is dependent upon the brain region, cell type and subcellular localization (Robbins and Tempel, 2012).

Heterozygous mutations in the *KCNA1* gene, encoding the Kv1.1 α subunit, were associated with episodic ataxia type 1 (EA1), a dominantly inherited disorder characterized by generalized ataxia attacks and spontaneous muscle quivering (Browne et al., 1994). Interestingly, a subset of patients with familial EA1 shows epileptic seizures, suggesting that Kv1.1 dysfunctions may play a role in the pathophysiology of epilepsy (Spauschus et al., 1999; Zuberi et al., 1999; Eunson et al., 2000). Loss-of function mutations reported in the *KCNA1* gene of EA1 patients cause reduced current amplitude thus contributing to seizures susceptibility (Browne et al., 1994; Adelman et al., 1995; D’Adamo et al., 1999; Imbrici et al., 2006).

In support of the hypothesis of an epileptogenic role of *KCNA1* mutations, several knock-out mouse models for this gene developed an epileptic phenotype (Smart et al., 1998; Rho et al., 1999). Biochemical and biophysical studies demonstrated a colocalization of Kv1.1 and Kv1.2 subunits in several subcellular brain regions and that they could form heteromeric channels, which are reported as profoundly altered by EA1 mutations (D’Adamo et al., 1999).

Notably, a Kv1.2 knock-out mouse model displayed increased seizure susceptibility (Brew et al., 2007). In this regard, Syrbe et al. (2015) recently identified *de novo* loss or gain-of-function mutations in *KCNA2* gene (**Table 2**), encoding the Kv1.2 channel, in patients showing mild to severe epileptic encephalopathy. A role of Kv1.2 was also suggested by another case report describing a *de novo* mutation, leading to the p.Arg297Gln amino acid substitution in a patient affected by ataxia and myoclonic epilepsy (Pena and Coimbra, 2015).

### Kv4

The Kv4 channels are highly expressed in the brain and mediate the main dendritic A-currents which critically regulate action potential back-propagation and the induction of specific forms of synaptic plasticity. In particular, the Kv4.2 subunit is a key component of the A-type potassium current in the CNS (*I*_A_) (Birnbaum et al., 2004).

In 2006, Singh and collaborators described a truncation mutation (p.Asn587fsX1) in the Kv4.2 channel encoded by the *KCND2* gene, in a patient affected by temporal lobe epilepsy (TLE). This mutation causes a frame-shift, leading to a premature termination codon and consequently to a Kv4.2 channel haploinsufficiency (Singh et al., 2006). Recently, a whole exome sequencing study identified a *de novo* gain-of-function mutation (p.Val404Met) in *KCND2.* The mutation was found in monozygotic twins affected by autism and severe intractable seizures and occurred at a highly conserved residue within the C-terminus of the S6 transmembrane region of the ion pore. A functional analysis of mutated channels revealed a significantly slowed channel inactivation (Lee et al., 2014).

Very recently, an involvement of Kv4.3 subunits in epilepsy was also suggested by the identification of a *de novo* mutation (p.Arg293_Phe295dup) in the relevant *KCND3* gene causing a severe channel dysfunction in a patient with complex early onset cerebellar ataxia, intellectual disability, oral apraxia and epilepsy. This mutation results in the duplication of a RVF (Arginine–Valine–Phenylalanine) motif in the S4 segment and leads to a more positively charged voltage-sensor domain, altering the voltage-dependent gating properties of the channel. In details, the p.Arg293_Phe295dup mutation induced a strong depolarizing shift in the voltage dependence of both the activation (about +59.3 mV) and inactivation (+62 mV) of the channel (Smets et al., 2015).

### Kv7

KCNQ (Kv7) channels are low-threshold activated voltage-gated potassium channels. Among the five known isoforms, KCNQ2–5 are expressed throughout the nervous system, whereas KCNQ1 is mostly expressed in cardiac tissue. The *KCNQ2* gene is the most commonly reported as mutated in epilepsy. Its mutations cause neonatal epilepsies with wide phenotypic heterogeneity, ranging from benign familial neonatal seizures (BFNS) with normal cognition and unremarkable neuroimaging to early onset epileptic encephalopathies (EOEEs) with mental retardation, suppression-burst electroencephalography (EEG) and distinct neuroradiologic features (Singh et al., 1998; Weckhuysen et al., 2012; Soldovieri et al., 2014). More than 80 different mutations in *KCNQ2*, consisting of missense, non-sense, truncations, splice site defects and frame-shift mutations, as well as sub-microscopic deletions or duplications, were described and most of them are found in the pore region and the large intracellular C-terminal domain (Lee et al., 2009). Functional studies suggested a strict phenotype/genotype correlation between disease severity and functional properties of mutant channels (Miceli et al., 2013). KCNQ2 is a primary player that mediates neuronal muscarinic (M) currents: the opening of this channel or of heterogeneous KCNQ2/KCNQ3 complexes inhibits initiation of action potential and thus suppresses neuronal excitability (Brown and Passmore, 2009).

Mutations in *KCNQ3* gene have been described in families affected with benign epilepsy with variable age of onset and good outcome (Zara et al., 2013; Grinton et al., 2015) or in a patient with benign childhood epilepsy with centrotemporal spikes (BECTS) (Fusco et al., 2015). However, two recent reports suggested that mutations in *KCNQ3*, similarly to *KCNQ2*, can be also found in patients with more severe phenotypes, including intellectual disability. In particular, they described *KCNQ3* mutations in patients with early onset epilepsy and neurocognitive deficits (Soldovieri et al., 2014; Miceli et al., 2015; **Table 2**).

Mutations in the *KCNQ1* gene were associated with a particular form of long QT syndrome, the LQT1 (Wang et al., 1996). Interestingly, some authors observed that epilepsy occurred in mouse lines bearing dominant human LQT1 mutations in this channel, which caused syncope and sudden death (Goldman et al., 2009). Moreover, genetic variants in the *KCNQ1* gene were reported in three cases of sudden unexpected death in epilepsy (SUDEP), a catastrophic complication of human idiopathic epilepsy with unknown causes. However, the relationship of these variants to the disease remains to be elucidated (Yang et al., 2009; Partemi et al., 2015). The evidence that *KCNQ1* genetic variations may confer susceptibility for recurrent seizure activity increasing the risk of sudden death is further supported by the description of a pathogenic *KCNQ1* variant (p.Leu273Phe) in a family featuring LQTS and epilepsy (Tiron et al., 2015).

### Kv8

The *KCNV2* gene encodes the voltage-gated K^+^ channel Kv8.2. This subunit is electrophysiologically silent when assembled in homotetramer. Otherwise, it significantly reduces the surface expression of the resulting channels and influences their biophysical properties when involved in the formation of functional heterotetramers with Kv2 subunits (Czirják et al., 2007). Kv2.1 and Kv8.2 show significant regional overlap: within the hippocampus, transcripts for both *KCNV2* and *KCNB1*, which encodes Kv2.1, are detected in excitatory neurons of the pyramidal cell layers and the dentate gyrus. Similarly, both of them are abundantly expressed in the cortex (Maletic-Savatic et al., 1995). Their regional colocalization is consistent with an effect of Kv8.2 variants on Kv2.1 channels within cells critically important for seizure generation and propagation.

A support of the involvement of *KCNV2* in seizure pathogenesis was provided by the identification of non-synonymous variants in two unrelated children showing epilepsy: p.Arg7Lys and p.Met285Arg. In particular, the p.Arg7Lys was found in a patient affected by febrile and afebrile partial seizures, whereas the p.Met285Arg was reported in a case of epileptic encephalopathy and severe refractory epilepsy. The functional characterization of these variants demonstrated that they both enhanced Kv8.2-mediated suppression of Kv2.1 currents, suggesting a role in decreasing delayed rectifier K^+^ current in neurons, therefore increasing cells excitability. Moreover, the p.Met285Arg caused a shift in the voltage-dependence of activation as well as slower activation kinetics, in accordance with the more severe clinical phenotype of the patient (Jorge et al., 2011).

### Kv11-HERG

The human ether-a-go-go-related gene (*hERG*, also known as *KCNH2*) encodes the pore-forming subunit of the rapid component of the delayed rectifier K^+^ channels, Kv11.1, which are expressed in several tissues, mostly in brain and heart. In the brain, Kv11.1 channels regulate neuronal firing and modulate the excitability of GABAergic and dopaminergic neurons. The same channel exerts a different function in the heart being involved in the regulation of membrane potentials in the ventricles (Vandenberg et al., 2012).

Mutations in the *KCNH2* gene were reported to cause type 2 long QT syndrome (LQT2), a rare inherited ion channel disorder characterized by prolonged QT interval and predisposing patients to ventricular arrhythmias that can lead to syncope and sudden cardiac death (SCD). LQT2 syndrome is frequently misdiagnosed as epilepsy due to seizures that are triggered by cerebral hypoperfusion during a ventricular arrhythmia, therefore suggesting a possible link between epilepsy and cardiac arrhythmias, as described by several clinical reports (Johnson et al., 2009; Keller et al., 2009; Omichi et al., 2010; Tu et al., 2011; Zamorano-León et al., 2012; Partemi et al., 2013). In particular, a seizure phenotype was reported in about 30% of unrelated LQTS patients carrying pathogenic variants in the *KCNH2* gene, suggesting that mutations in the Kv11.1 channel associated with LQTS may also predispose to seizure activity (Johnson et al., 2009). Moreover, a post-mortem study identified nearly 13% of LQTS pathogenic variants in the *KCNH2* and *SCN5A* genes in epileptic samples. In particular, regarding *KCNH2*, two non-synonymous mutations have been identified: p.Arg176Trp and p.Arg1047Leu (Tu et al., 2011). Another study on three families showing a history of seizures and LQTS2 lead to the identification of three novel *KCNH2* mutations: p.Tyr493Phe, Ala429Pro and Thr74ArgfsTer32 (also named p.del234-241). *In vitro* functional analyses of all these variants showed a loss of hERG potassium channel function with a reduction of the current, suggesting a dominant negative effect (Keller et al., 2009). Omichi et al. (2010) reported a case of a man with long history of epilepsy and referred for cardiologic evaluation, showing the p.Arg534Cys mutation. In addition, other authors identified a nonsense mutation (p.Arg863X) leading to a 296-amino acid deletion (Zamorano-León et al., 2012) while a loss-of-function mutation (p.Ile82Thr) was reported in a pedigree featuring LQTS, idiopathic epilepsy and increased risk of sudden death (Partemi et al., 2013).

## Auxiliary Subunits of Kv Channels

Kv channel functional diversity is enhanced by coassembly with a wide array of auxiliary subunits, which cannot form functional channels alone but which can greatly impact channel function upon coassembly with α-subunits to form hetero-oligomeric complexes (Trimmer, 1998). Defects in these subunits may affect Kv channel function and network excitability, resulting thus in an increase of seizure susceptibility. Several subunits have been identified, including β-subunit (Kvβ), leucine-rich glioma-inactivated-1 (Kv_LGI1_) and K^+^ channel-interacting protein (Kv_KChIP_).

### Kvβ

Kvβ subunits are cytoplasmatic proteins critical for the correct membrane localization and normal biophysical properties of voltage-gated K^+^ channels. Variations in the expression of different Kvβ genes and their isoforms could significantly impact K^+^ channel function, especially with respect to inactivation kinetics. In the mammalian genome three genes encode Kvβ subunits: Kvβ1, Kvβ2, and Kvβ3 (Pongs and Schwarz, 2010). Interestingly, Kvβ2 knockout mouse models were characterized by cold-swim induced tremors and occasional seizures, suggesting thus a role of this subunit in the regulation of neuronal excitability (McCormack et al., 2002). An association between the severity of seizures and the loss-of-function of the *KCNAB2* gene that encodes the β2 subunit was reported (Heilstedt et al., 2001). In particular, the hemizygous deletion of *KCNAB2* identified in this manuscript in epileptic patients suggested that haploinsufficiency of this gene may represent a significant risk factor for epilepsy: the lack of the β subunit would reduce K^+^ channel-mediate membrane repolarization and increase neuronal excitability (Heilstedt et al., 2001).

### Kv_LGI1_

The leucine-rich glioma-inactivated-1 (LGI1) is the best characterized LGI family protein, highly expressed in neurons, which encodes a secreted protein containing two domains [a leucine-rich repeat domain (LRR) and a β-propeller domain called EPTP] that mediate protein-protein interactions. LGI1 binds to the presynaptic voltage-gated potassium channel Kv1.1 and prevents Kv channel inactivation mediated by the β subunit of the channel (Schulte et al., 2006). The *LG1* gene was find to be mutated in approximately 50% of ADLTE (autosomal dominant lateral TLE) families: more than 30 disease-causing mutations in *LGI1* gene have been associated so far with this focal epilepsy that is characterized by good response to antiepileptic drugs and with a juvenile onset (Kalachikov et al., 2002; Morante-Redolat et al., 2002; Dazzo et al., 2015). In particular, almost all mutations are missense, splice-site, or short indels (Nobile et al., 2009; Ho et al., 2012) while only a single microdeletion has been reported (Fanciulli et al., 2012). Certain LGI1 mutants (typically non-secreted mutants) fail to prevent channel inactivation resulting in more rapidly closing channels, which extends presynaptic depolarization and leads to increased calcium (Ca^2+^) influx. Consequently, release of neurotransmitter is increased excessively and may induce focal seizures (Nobile et al., 2009). Moreover, it was demonstrated that the loss of *LGI1* gene in mice induced lethal epilepsy, suggesting its essential role as an antiepileptogenic ligand. LGI1 may serve as a major determinant of brain excitation and the LGI1 gene-targeted mouse could provide a good model for human epilepsy (Fukata et al., 2010).

### Kv_KChIP_

The K^+^ channel-interacting proteins (KChIPs 1–4) compose a subfamily of neuronal Ca^2+^ sensor proteins that modulate trafficking, targeting to the plasma membrane, as well as turnover and endocytosis of Kv4 channels (An et al., 2000). Among KChIPs, KChIP2 is abundantly expressed in hippocampal pyramidal cells and represents the major target of Kv4 α subunits to form a complex essential for *I*_A_ regulation in hippocampal neurons (Rhodes et al., 2004). This current has been found to be reduced in the presence of a deletion in the *KChIP2* gene by Wang and collaborators. The authors thus suggested that it may increase susceptibility to seizures (Wang et al., 2013). Moreover, they also hypothesized a role of *KChIP2* in SUDEP risk (Wang et al., 2013), since *KChIP2* knockout mice were previously shown to be highly susceptible to induced arrhythmias (Kuo et al., 2001). In conclusion, these data suggested that loss-of-function mutations in modulatory subunits could increase the susceptibility to seizures and cardiac arrhythmias, thereby providing a unified mechanism for a neurocardiac syndrome such as SUDEP.

## Inwardly Rectifying Potassium Channels

Inwardly rectifying K^+^ (Kir) channels are widely expressed in several excitable and non-excitable tissues playing a key role in the maintenance of the resting membrane potential and consequently in the regulation of cell excitability. Approximately 15 Kir clones forming either homotetramers or heterotetramers were identified and grouped in seven different families based on sequence similarity and functional properties: Kir1-Kir7 (Hibino et al., 2010). Generally, Kir channels showed the greater conductance at negative potentials in respect to the equilibrium potential for K^+^ (*E*_K_), while an inhibition of the outward flow of K^+^ ions caused by both Mg^2+^ and polyamines was reported at more positive values (Lopatin et al., 1994). Several Kir channels have been associated with epileptic phenotypes and, in particular, Kir2.1, Kir3, Kir4 and Kir6.

### Kir2.1

The Kir2.1 channel is encoded by the *KCNJ2* gene whose expression is reported in several brain areas (Karschin et al., 1996) as well as in astrocytes where they control astrocyte-mediated K^+^ buffering in combination with Kir4.1 (Jabs et al., 2008; Chever et al., 2010).

Several mutations impairing the channel functionality were reported in the *KCNJ2* of Andersen–Tawil syndrome (ATS) patients (Haruna et al., 2007; Chan et al., 2010; Guglielmi et al., 2015; see **Table 2** for mutation details). On the other hand, Kir2.1 gain-of-function mutations cause the type-3 variant of the short QT syndrome (SQT3s) which results in QT shortening and increased risk of sudden cardiac death (Priori et al., 2005). Recently, some authors detected a novel mutation (p.Lys346Thr) in the *KCNJ2* in monozygotic twins displaying SQT3s and autism-epilepsy phenotype, suggesting the existence of a Kir2.1 role in neuropsychiatric disorders and epilepsy. Functional studies revealed that this mutation causes an increase of the channel’s surface expression and stability at the plasma membrane, a reduction in protein degradation and an altered protein compartmentalization (Ambrosini et al., 2014).

### Kir3-GIRK

The G-protein-coupled Kir (GIRK) channels belong to the subfamily of Kir3 that are important regulators of electrical excitability in both cardiomyocytes and neurons (Slesinger et al., 1995). Different types of neurotransmitters, such as acetylcholine, dopamine, opioids, serotonin, somatostatin, adenosine, and GABA, activate these channels by stimulating their G-protein coupled receptors (GPCRs). This results in a final membrane hyperpolarization and inhibition of cell excitability due to the activation of an outward flux of K^+^ ions (Krapivinsky et al., 1995; Slesinger et al., 1995). Mammals express four GIRK channel subunits (GIRK1-4, also named Kir3.1-3.4), encoded by *KCNJ3*, *KCNJ6*, *KCNJ9*, and *KCNJ5*, respectively. These four subunits can form homo or heterotetramers with unique biophysical properties, regulation and distribution (Lüscher and Slesinger, 2010).

Alterations in GIRK channel function have been associated with pathophysiology of severe brain disorders, including epilepsy. In this regard, a GIRK2 knockout mouse model resulted to be more susceptible to develop both spontaneous or induced seizures in respect to wild type mice (Signorini et al., 1997). In particular, mice carrying a p.Gly156Ser mutation displayed an epileptic phenotype (Patil et al., 1995). Indeed, this mutation has been found to alter the putative ion-permeable, pore-forming domain of the channel, inducing Ca^2+^ overload in cells and reducing channel availability, leading thus to neurodegeneration and seizures susceptibility (Slesinger et al., 1996).

An increased expression of GIRK channels was observed in rat brain after an electroconvulsive shock, probably altering the excitability of granule cells and the functions of neurotransmitter receptors which are coupled to these channels (Pei et al., 1999). Another evidence in support of a role of GIRK channels in epilepsy was provided by the demonstration that ML297, a potent and selective activator of GIRK channels, showed epileptogenic properties in mice (Kaufmann et al., 2013). On the other hand, the inhibition of GIRK channel activity by drugs causes seizures (Mazarati et al., 2006). All these considerations imply that changes in Kir3 channel activity may alter the susceptibility to seizures.

### Kir4

Among Kir4 channels, the Kir4.1, encoded by the *KCNJ10* gene, is the only one that has been associated to epilepsy. This subunit can assemble itself in homomeric channels or it can constitute heterotetramers in combination with Kir5.1 (*KCNJ16*) (Pessia et al., 2001). Kir4.1 expression has been detected primarily in the thalamus, cortex, brainstem and hippocampus (Higashi et al., 2001). Kir4.1 channels play a key role in maintaining resting membrane potential by transporting K^+^ from the extracellular space into glial cells in the CNS (Nishida and MacKinnon, 2002).

Alterations of Kir4.1 channels have been linked to seizure susceptibility in both mice (Ferraro et al., 2004) and humans (Buono et al., 2004). Conditional Kir4.1 knockout mice in astrocytes have been found to display premature lethality and severe seizures prior to death (Djukic et al., 2007), supporting the idea of a pathophysiological relationship of the Kir4.1 impairment with epilepsy. Concerning human Kir4.1, a linkage study identified a missense variation (p.Arg271Cys) as associated with epileptic phenotypes (Buono et al., 2004). However, the variant did not result to have functional effects *in vitro* (Shang et al., 2005). Mutations in this gene were also reported in EAST syndrome (also named SeSAME) patients, a rare condition showing epileptic seizures among other signs (Bockenhauer et al., 2009; Scholl et al., 2009; Freudenthal et al., 2011; see **Table 2** for mutation details).

Single nucleotide variations in Kir4.1 were detected in the DNA of TLE patients presenting with hippocampal sclerosis and antecedent febrile seizures, supporting the importance of *KCNJ10* as a candidate gene for seizures susceptibility (Heuser et al., 2010).

Interestingly, several authors reported a strong association between epilepsy and autism spectrum disorders (ASDs) and an “autism-epilepsy phenotype” has been proposed (Tuchman et al., 2009; Lee et al., 2015). Indeed, a mutational screening of *KCNJ10* in 52 children affected by cryptogenic epilepsy identified two heterozygous mutations (p.Arg18Gln and p.Val84Met) in three children of two unrelated families displaying seizures, ASDs, and intellectual disability. The functional consequences of these mutations appeared to be a gain-of-function mechanism. These findings suggest that an abnormal K^+^ homeostasis in the brain may increase the susceptibility to this “autism-epilepsy phenotype” (Sicca et al., 2011). A common mechanism between autism and epilepsy could be the impairment of astrocytic-dependent K^+^ buffering, altering neuronal excitability and synaptic function.

### Kir6-K_ATP_

The adenosine triphosphate (ATP)-sensitive K^+^ (K_ATP_) channels are widely distributed in various tissues where they couple cell metabolism to cell excitability. These channels are assembled by an inward rectifier K^+^ channel pore (Kir6.1/Kir6.2) and an ATP-binding regulatory subunit, named sulfonylurea receptor (SUR1/SUR2A/SUR2B) (Olson and Terzic, 2010). Neuronal K_ATP_ channels are mainly constituted by a coassembly of Kir6.2/SUR1 subunits. (Inagaki et al., 1995).

Several gain-of-function mutations were detected in the Kir6.2 (*KCNJ11*) or the SUR1 subunit (*ABCC8*). These mutations are responsible for developmental delay, epilepsy and neonatal diabetes (DEND), accounting for approximately 40% of cases and caused a decrease in the ability of ATP to block the K_ATP_ channel. This results in more fully openings of the channel at physiologically relevant concentrations of ATP, thus increasing the K_ATP_ current (Hattersley and Ashcroft, 2005). Nevertheless, the pathophysiological mechanism leading to epilepsy remains to be elucidated. Probably, elevated levels of extracellular glucose and intracellular ATP attenuate K_ATP_ channels, producing a more excitable state (Huang et al., 2007). Moreover, mice lacking Kir6.2 are vulnerable to hypoxia, exhibiting a reduced threshold for generalized seizure (Yamada et al., 2001). Transgenic mice, overexpressing the SUR1 gene in the forebrain, show a significant increase in the threshold for kainate-induced seizures (Hernández-Sánchez et al., 2001).

## Sodium-Activated Potassium Channels (K_Na_)

The Na^+^-activated K^+^ channels (K_Na_) are found in neurons throughout the brain and are responsible for delayed outward currents named *I*_KNa_. These currents regulate neuronal excitability and the rate of adaption in response to repeated stimulation at high frequencies. In many cases, *I*_KNa_ is mediated by the phylogenetically related K_Na_ channel subunits Slack and Slick (Bhattacharjee and Kaczmarek, 2005). Like the Kv channels, these subunits have six hydrophobic, transmembrane segments (S1–S6) with a pore P-domain between S5 and S6 and a large cytoplasmatic C-terminal domain containing two regulators of K^+^ conductance (RCK) domains that are likely to be sites for Na^+^-binding and channel gating. The Slack subunit binds with Slick to form heterotetrameric channel complexes (Kaczmarek, 2013). Slack has been associated with different epilepsy phenotypes.

## Slack

The *KCNT1* gene encodes the K_Na_ channel subunit KCNT1, called Slack (sequence like a calcium-activated potassium channel, also known as K_Ca4.1_ or Slo2.2). *KCNT1* is highly expressed in the brain but also in the heart and the kidney at lower levels. Concerning brain, it is not widely expressed in the cortex but it is found in neurons of the frontal cortex (Bhattacharjee et al., 2002), consistent with its known role in the pathogenesis of autosomal dominant nocturnal frontal lobe epilepsy (ADNFLE) (Heron et al., 2012). While KCNT1 channels are thought to play important roles in modulating the firing patterns and general excitability of many types of neurons, their precise function is yet to be resolved.

Mutations in *KCNT1* gene have been found in different epilepsy syndromes: ADNFLE (Heron et al., 2012; Kim et al., 2014; Møller et al., 2015), epilepsy of infancy with migrating focal seizures (EIMFS, previously known as malignant migrating partial seizures in infancy, MMPSI or also more recently as malignant migrating focal seizures of infancy, MMFSI) (Barcia et al., 2012; Ishii et al., 2013; Ohba et al., 2015; Rizzo et al., 2016) and other types of EOEEs, (Vanderver et al., 2014; Ohba et al., 2015), including Ohtahara syndrome (OS) (Martin et al., 2014). The involvement of *KCNT1* in these distinct disorders suggests that *KCNT1* mutations may cause a spectrum of focal epilepsies (Møller et al., 2015). Patients displaying *KCNT1* mutations have a very high occurrence of severe mental and intellectual disability.

Four missense mutations (p.Arg398Gln, p.Tyr796His, p.Met896Ile, and p.Arg928Cys) in *KCNT1* gene were reported to be associated with ADNFLE cases showing comorbidities of intellectual disability and psychiatric features (Heron et al., 2012). This is in contrast to ADNFLE patients without mutations in *KCNT1* gene, where intelligence and other neurologic functions are largely unimpaired (Phillips et al., 1998). Mutations are clustered around the RCK and cytoplasmatic NAD^+^ binding domain (Heron et al., 2012), the site that regulates the channel sensitivity to Na^+^ intracellular concentrations (Tamsett et al., 2009). A complete penetrance is reported in ADNFLE families showing *KCNT1* mutations (Heron et al., 2012) with the exception of a non-penetrant case (Møller et al., 2015).

Interestingly, Møller et al. (2015) reported that a *KCNT1* mutation (p.Arg398Gln) can lead to either ADNFLE or EIMFS within the same family, indicating that genotype-phenotype correlations are not straightforward). Similarly, a more recent study showed that the p.Gly288Ser mutation could cause both phenotypes, probably due to genetic modifiers or environmental factors (Kim et al., 2014). Nevertheless, this association was unexpected since *in vitro* studies demonstrated that mutations associated with MMFSI caused a significantly larger increase in current amplitude than those associated with ADNFLE (Milligan et al., 2014).

Concerning EIMFS, in addition to the above mentioned p.Gly288Ser and p.Arg398Gln, several additional mutations have been identified, including p.Val271Phe, p.Arg428Gln, p.Arg474Gln, p.Met516Val, p.Lys629Asn, p.Ile760Met, p.Pro924Leu and p.Ala934Thr (Barcia et al., 2012; Ishii et al., 2013; Mikati et al., 2015; Ohba et al., 2015; Rizzo et al., 2016). These are clustered not only around the RCK and NAD^+^ binding domain of the protein, but also within its S5 transmembrane segment, indicating that the alteration of other regions of KCNT1 could also be pathogenic (Ishii et al., 2013; McTague et al., 2013; Kim et al., 2014)

Finally, two *KCNT1* mutations were associated with other forms of EOEEs, strengthening once again the existence of a wide phenotypic spectrum of *KCNT1* mutations. In particular, the p.Phe932Ile was detected in a patient affected by EOEEs whereas the p.Ala966Thr was found in one showing OS. Both of them are clustered around the RCK and NAD^+^ binding domains of the protein (Martin et al., 2014; Vanderver et al., 2014; Ohba et al., 2015).

The effect of nine different mutations in *KCNT1* gene that give rise to these distinct forms of epilepsy was examined and it was demonstrated that they all result in channels displaying a strong gain-of-function phenotype: all of them produced many-fold increases in current amplitude as compared with the wild-type channel. This could greatly increase the cooperativity in channel gating that is detected in clusters of multiple channels (Kim et al., 2014).

## Calcium-Activated Potassium Channels (K_Ca_)

Ca^2+^-activated K^+^ channels are highly conserved complexes thought to play a critical role in neuronal firing properties and circuit excitability in the human brain. Three groups of Ca^2+^-activated K^+^ channels can be distinguished: large conductance (BK_Ca_), intermediate conductance (IK_Ca_), and small conductance (SK_Ca_) channels (N’Gouemo, 2011). The opening of these channels is in response to an increase in Ca^2+^ concentration and a depolarization of the membrane potential, which in turn causes a secondary hyperpolarization reestablishing the membrane potential as well as Ca^2+^ levels. Otherwise it can produce an afterhyperpolarization to potentials more negative than the resting membrane potential (Latorre and Brauchi, 2006; Nardi and Olesen, 2008). To date, only the association between K_Ca1.1_ channel and epilepsy has been demonstrated.

### K_Ca1.1_

*KCNMA1* gene encoded the α-subunit of the large conductance K_Ca1.1_ channels. They show the typical tetrameric structure of K^+^ channels, with four α-subunits each displaying seven transmembrane segments, with a unique S0 segment, and the charged S4 segment conferring the voltage-dependence. Ca^2+^ sensitivity comes instead from the bulky C-terminal tail that includes a negatively charged, high-affinity Ca^2+^ binding region (Jiang et al., 2001) and the double negative charged RCK-domain. These channels could associate with four different types of β subunits (β1-β4, each encoded by a specific gene *KCNMB1-4*) which modulated channel function uniquely (Orio et al., 2002).

K_Ca1.1_ channels play a role in promoting high neuronal frequency firing which is consistent with their predominant expression in axon and presynaptic terminals of neurons located in brain regions (e.g., hippocampus and cortex) frequently involved in epilepsy (Gu et al., 2007; Martire et al., 2010). The involvement of these channels in epilepsy was suggested not only by their localization but also by studies on animal models. In this regard, it has been demonstrated in mice highly susceptible to convulsions that the inhibition of K_Ca1.1_ channels is sufficient to block cortical bursting activity (Jin et al., 2000). Moreover, the loss of β4 subunits in K_Caβ4_ knockout mice promoted the excitatory synaptic transmission, resulting in temporal cortex seizures (Brenner et al., 2005). Finally, Ermolinsky et al. (2008) demonstrated a deficit of *KCNMA1* expression in the dentate gyrus in animal models, hypothesizing therefore its critical role in the pathogenesis of mesial temporal lobe epilepsy (mTLE).

An association between K_Ca1.1_ channels and epilepsy has also been observed in humans. A missense mutation in *KCNMA1* (p.Asp434Gly) was detected in a large family with generalized epilepsy and paroxysmal dyskinesia. Functional studies revealed an increased Ca^2+^ sensitivity predicting a gain-of-function and neuronal hyperexcitability by a presumably faster action potential repolarization (Du et al., 2005). Additional studies suggested that depending on the distribution of the various β subunits in the brain, this mutation can differently modulate K_Ca1.1_ channels contributing to the pathophysiology of epilepsy and dyskinesia (Lee and Cui, 2009). As far as genes different from *KCNMA1*, a polymorphism in *KCNMB4*, named rs398702, was also associated with mTLE in an Irish cohort population (Cavalleri et al., 2007) but the study failed to be replicated (Manna et al., 2013), while a truncation mutation in *KCNMB3* (p.Val256TyrfsTer4) affecting synaptic inhibition and thereby increasing neuronal excitability and seizure susceptibility, was associated with idiopathic generalized epilepsy (Hu et al., 2003; Lorenz et al., 2007).

## Concluding Remarks

Epilepsy is one of the most common chronic and heterogeneous neurological disorders, affecting 1–2% of the population, characterized by recurrent unprovoked seizures due to abnormal synchronized electrical discharges within the CNS (Steinlein, 2004). Since ion channels mediate the axonal conduction of action potentials and transduction through synaptic transmission, increasing evidence suggests that any mutation-induced channel malfunction directly alter brain excitability and can induce epileptic seizures. Therefore, the discovery of genetic defects and, in particular, the electrophysiological characterization of mutant ion channels in hereditary forms of epilepsy may elucidate pathophysiological concepts of hyperexcitability in the CNS. This knowledge could enable new therapeutic strategies by antagonizing the epilepsy-causing mechanisms using the defective proteins as pharmacological targets. Given these considerations, we present an overview of mutations in K^+^ channels and their related accessory subunits underlying different human epileptic phenotypes. Several families of K^+^ channels have been involved in the pathogenesis of epilepsy or other syndromes showing seizures as a clinical sign. For each channel family, the effect of reported mutations is different: loss-of-function as well as gain-of-function could be observed. The common effect of all mutations is to determine membrane hyperexcitability, thus increasing the susceptibility to seizures. Our review highlights the pleiotropic effects of some mutations in K^+^ channels and the lack of a direct genotype–phenotype correlation. Interestingly, K^+^ channels dysfunctions seem to be mainly observed in epileptic patients with neurological comorbidities, such as ASDs, intellectual disabilities or psychiatric features, in which they are associated with more clinical severity. This observation could suggest to perform a mutation screening of K^+^ channels in patients showing intellectual disabilities.

In conclusion, the discovery of K^+^ channels encoding genes that influence susceptibility and disease progression will provide insight into the molecular events of epileptogenesis, improve molecular diagnostic utility, and identify novel therapeutic targets for treatment of human epilepsy.

## Author Contributions

All authors listed, have made substantial, direct and intellectual contribution to the work, and approved it for publication.

## Conflict of Interest Statement

The authors declare that the research was conducted in the absence of any commercial or financial relationships that could be construed as a potential conflict of interest.
